# Homozygous deletion of *TNFRSF4, TP73, PPAP2B* and *DPYD* at 1p and *PDCD5* at 19q identified by multiplex ligation-dependent probe amplification (MLPA) analysis in pediatric anaplastic glioma with questionable oligodendroglial component

**DOI:** 10.1186/1755-8166-7-1

**Published:** 2014-01-06

**Authors:** Miguel Torres-Martín, Carolina Peña-Granero, Fernando Carceller, Manuel Gutiérrez, Rommel R Burbano, Giovanny R Pinto, Javier S Castresana, Bárbara Melendez, Juan A Rey

**Affiliations:** 1 Molecular Neuro-oncogenetics Laboratory, Research Unit-Unidad de Investigación, Hospital Universitario La Paz, Paseo de la Castellana 261, 28046 Madrid, Spain; 2 Department of Pediatric Neurosurgery, Hospital Universitario La Paz, Paseo de la Castellana 261, 28046 Madrid, Spain; 3 Department of Pathology, Hospital Universitario La Paz, Paseo de la Castellana 261, 28046 Madrid, Spain; 4 Institute of Biological Sciences, Human Cytogenetics Laboratory, Federal University of Para, R Augusto Correa 01, Belem, PA 66075-110, Brazil; 5 Genetics and Molecular Biology Laboratory, Federal University of Piau, Avda Sao Sebastiao 2819, Parnaiba, PI 64202-020, Brazil; 6 Brain Tumor Biology Unit, University of Navarra School of Sciences, Irunlarrea 1, 31008 Pamplona, Spain; 7 Molecular Pathology Research Unit, Virgen de la Salud Hospital, Avda Barber 30, 45004 Toledo, Spain

**Keywords:** Pediatric anaplastic glioma, Oligodendroglioma, Homozygous deletion 1p/19q, MLPA

## Abstract

**Background:**

Pediatric oligodendrogliomas are rare and appear to show a different molecular profile from adult tumors. Some gliomas display allelic losses at 1p/19q in pediatric patients, although less frequently than in adult patients, but this is rare in tumors with an oligodendroglial component. The molecular basis of this genomic abnormality is unknown in pediatric gliomas, but it represents a relatively common finding in pediatric oligodendroglioma-like neoplasms with leptomeningeal dissemination.

**Results:**

Multiplex ligation-dependent probe amplification (MLPA) analysis using SALSA P088-B1 for the analysis of the 1p/19q allelic constitution in a pediatric anaplastic (oligodendro)-glioma showed homozygous co-deletion for markers: *TNFRSF4* (located at 1p36.33), *TP73* (1p36.32), *PPAP2B* (1pter-p22.1), *DPYD* (1p21.3), and *PDCD5* (19q13.12), and hemizygous deletion of *BAX* (19q13.3-q13.4). No sequence changes for R132 and R172 of the *IDH1/2* genes were identified.

**Conclusions:**

The molecular findings in this pediatric anaplastic glioma do not allow for a clearly definitive pathological diagnosis. However, the findings provide data on a number of 1p/19q genomic regions that, because of homozygotic deletion, might be the location of genes that are important for the development and clinical evolution of some malignant gliomas in children.

## Background

According to Hargrave D [1] high grade gliomas in pediatric oncology generally group grade III and IV tumors with astrocytic or oligodendroglial nature, and include anaplastic astrocytomas (grade III), glioblastomas (grade IV) and anaplastic tumors (grade III) that have a major oligodendroglial component, i.e., pure oligodendrogliomas and mixed oligo-astrocytomas. Although malignant astrocytomas represent approximately 8%-10% of all pediatric CNS tumors, most arising in the supratentorial region, high-grade oligodendrogliomas in children are rare [2]. An incidence of 6% has been reported for oligodendroglial tumors (including pure oligodendrogliomas and mixed oligo-astrocytomas) in children aged 0–14 years [2]. The histology of these neoplasms with oligodendroglial component is classical and has been described as having a “fried-egg” appearance with “chicken-wire” vasculature. Anaplastic variants are primarily based on the presence of mitotic activity, microvascularization and necrosis, while anaplastic astrocytomas are diffusely infiltrating tumors with increased cellularity, distinct nuclear atypia and marked mitotic activity [3,4]. The molecular biology of adult malignant gliomas is now well defined for tumors with either astrocytic or oligodendroglial characteristics. Both types of gliomas are very complex and genetically heterogeneous, with multiple alterations in critical pathways, primarily alterations of *MGMT* (methylation), *IDH1/2* (mutation), 1p/19q co-deletion, *EGFR* and *PI3K* pathway variations, and *p53* or *Rb* pathway mutation [5,6]. Among the pediatric malignant gliomas, *EGFR* gene amplification appears to occur less frequently than in tumors from adult patients, and only 2% have the EGFRvIII variant. In contrast, it appears that *PDGFRA* and *PDGFRB* are more commonly affected in high-grade pediatric gliomas [1,7,8]. Recently, whole genome sequencing in low-grade pediatric gliomas has identified multiple genetic alterations involving several genes such as *BRAF, RAF1, FGFR1, MYB* and *MYBL1*[9,10]. Copy number alteration analysis has demonstrated gains of chromosomes 7, 8 and 5q and loss of 1p [10]. Up to 6 significantly recurrent regions of focal deletion have been identified: 9p21.3 (and the adjacent region), 6q26, 10q21.3, 8p22 and 13q31.3, where multiple genes with functions related to cancer development are located [10]. In this report we describe the partial concurrent homozygous deletion at 1p/19q in an anaplastic glioma (or oligodendroglioma) that took place in a 6-year-old boy. This event might provide insights on a subgroup of pediatric (oligodendro)-gliomas with 1p/19q involvement.

## Case presentation

### Medical history and examination

A 6-year-old boy was admitted to the La Paz Hospital for complaining of tremors in the left arm. The patient had a history of occasional holocranial headaches and vomiting over the last 2 months and was experiencing thalamic pain. There were no memory or behavioral changes or performance impairment, and the examination revealed no papilloedema. The patient had a postural tremor (6–8 Hz) in the outstretched left arm, which was more prominent in the distal muscle groups. The tremor was slightly accentuated when moving the arm. The tremors ceased when the limb was at rest and when the patient was asleep. The patient had no nystagmus or gait ataxia, and his speech was normal. His osteo-cutaneous reflexes were slightly enhanced on the left side, but he had no sensory abnormalities. The examination revealed a slight left hemiparesis. The patient’s left hand was closed due to dystonia, and his left foot presented dystonia when walking.

**Figure 1:**
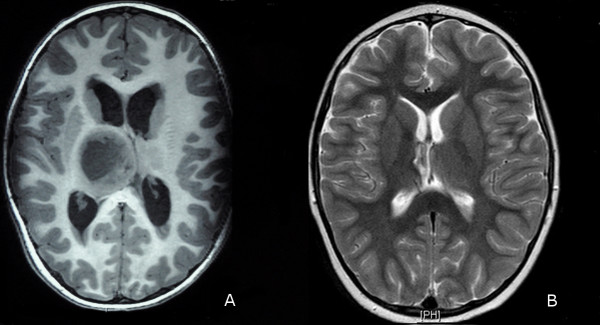
**MRI of pediatric glioma. (A)** MRI shows a large right thalamic tumor compressing the third ventricle. **(B)** MRI control 9 months later.

A brain CT scan revealed obstructive hydrocephalus caused by a hypo-dense right thalamic and upper brainstem tumor, which was producing a mass effect on the basal ganglia and a shift in the lateral ventricle. T1-weighted 3 T-MR imaging showed a large heterogeneous solid, cystic tumor arising from the right thalamus and extending to the brainstem. An MR-imaging study performed with gadolinium (Gd) contrast showed a ring-enhancing pattern. Diffusion-tensor imaging was conducted to locate the posterior limb of the internal capsule (PLIC) in the preoperative MR (Figure 1A). The laterally displaced PLIC was even more posterior than expected, which made an anterior approach the most appropriate.

### Surgery

The child underwent a right frontal craniotomy, and a trans-cortical frontal approach to the right lateral ventricle was performed for a gross total resection (GTR) of the tumor. This procedure was accomplished using a micro-neurosurgical technique. At the completion of the resection, an endoscopic third ventriculostomy was performed. An intraoperative pathological examination identified an anaplastic (possibly astrocytic) glioma.

### Postoperative course

The patient’s postoperative recovery was uneventful. There was a dramatic improvement in his symptoms in the immediate postoperative period with virtual cessation of the tremors. The initial follow-up using 3 T-MR images obtained 1 month after the surgery showed a GTR of the tumor and no hydrocephalus. After the initial follow-up, the child was treated with radiotherapy and chemotherapy. An MR image obtained 9 months later revealed no tumor recurrence, and the patient remained neurologically intact (Figure 1B). No changes have been observed after 2 years of follow-up.

### Anatomical pathology

**Figure 2:**
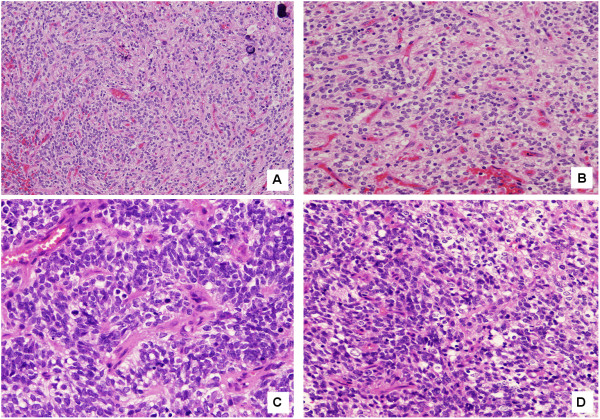
**Histopathological features of the pediatric glioma reported. (A)** Microcalcifications. **(B)** A prominent micro-vascular stroma presenting branching capillaries with endothelial tumefaction can be observed. **(C)** Anaplastic aspect with high nuclear to cytoplasmic ratio and mitosis was present. **(D)** Intermingled “egg-fried” cells can be observed in a number of anaplastic foci.

**Figure 3:**
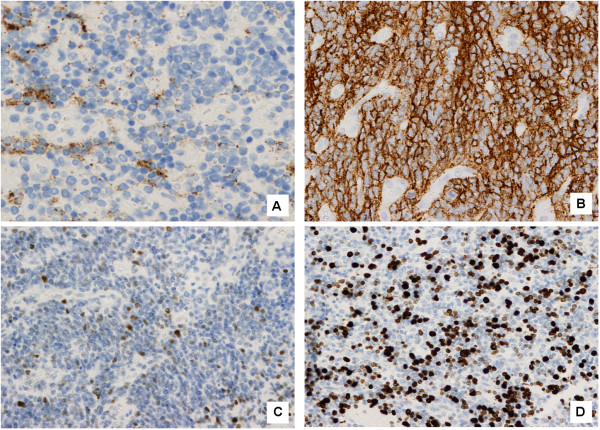
**Tumor immunoreactivity. (A)** GFAP immunoreactivity was variable whereas Synaptophysin was negative. **(B)** Cytoplasmic immunoreactivity for Vimentin and CD57 (HNK-1) were positive. **(C)** Nuclear p53 was positive in 15%-20% of cells. **(D)** Growth fraction (ki 67) was approximately 30%.

Tumor proliferation consisted of round to slightly elongated and densely packed cells with round or ovoid hyper-chromatic nuclei and scarce cytoplasm, occasionally expressed as perinuclear halos but without a characteristic honeycomb pattern. Micro-calcifications were present but there was no necrosis (Figure 2A). A prominent micro-vascular stroma presenting branching capillaries with endothelial tumefaction was observed (Figure 2B). Most tumor cells had an anaplastic aspect with a high nuclear to cytoplasmic ratio, and mitosis were present (Figure 2C). However, intermingled “fried-egg” cells were observed in a number of anaplastic foci (Figure 2D). GFAP immunoreactivity was variable whereas Synaptophysin was negative (Figure 3A). Cytoplasmic immunoreactivity for Vimentin and CD57 (HNK-1) were positive (Figure 3B), and. nuclear p53 was positive in 15%-20% of cells (Figure 3C). The growth fraction (ki 67) was approximately 30% (Figure 3D).

The pathology diagnosis was anaplastic glioma, although a 1p/19q analysis was suggested to obtain clues as to the presence of a possible oligodendroglial component. The unconventional microscopic pattern and the infrequent presentation of oligodendrogliomas in pediatric patients [2,3] could, however, justify our generic diagnosis.

## Methods

### Samples and DNA preparation

Samples and clinical-pathological data were obtained according to the hospital ethics committee procedures. DNA was isolated from frozen tumor and peripheral blood using the Wizard Genomic DNA purification kit (Promega). DNA from an oligodendroglioma with known 1p/19q loss was used as a positive control, and DNA from four healthy volunteers were used as references for multiplex ligation-dependent probe amplification (MLPA) reactions.

### Multiplex ligation-dependent probe amplification (MLPA) analysis

MLPA is a technique by which up to 45 different sequences corresponding to specific known genes (or genomic regions) can be targeted in a single, semi-quantitative polymerase chain reaction (PCR)-based experiment [11]. The sequences detected can be small (~60 nucleotides), enabling analysis of fragmented DNA. We used the MLPA SALSA P088-B1 kit (MRC-Holland, Amsterdam, Netherlands), which contains 15 1p probes, eight 19q probes, and 20 control probes specific to other chromosomes, including three 1q probes and two 19p probes. Information regarding the probe sequences and ligation sites can be found at http://www.mlpa.com. MLPA analysis was performed as described previously [12,13]. In brief, we used 100 ng for each normal DNA controls and tumor sample. DNA denaturation and hybridization of the SALSA probes was followed by a ligation reaction and PCR. One microliter of the amplified sample product was analyzed using the ABI 3100 Avant sequencer (Applied Biosystems) using the ROX-500 Genescan (ABI 401734) as an internal size standard. Duplicate experiments were performed to obtain accurate MLPA values (Table 1). Data analysis was performed with MRC-Coffalyser version 9.4 software (MRC-Holland, Amsterdam, Netherlands). Successful ligation reaction and identification of samples with insufficient amounts of DNA were verified using MLPA's internal ligation-independent probes. Normalization for tumor data (including positive control oligodendroglioma and pediatric patient sample), was performed on the average of the four references probes. Single regression for references and tumor data slope correction was performed. Given that a value of 1 corresponds to a normal allele constitution, LOH ratio limit was set at <0.67, homozygous loss at <0.34, and gains at >1.3. Probes were considered as altered only when duplicated runs corresponded to the same pattern.

**Table 1:** Summary of run by Coffalyser

**Gene**	**Markers**	**P Run1**	**P Run2**	**PC Run1**	**PC Run2**
*NOTCH2*	01p12	0.9	0.82	*0.57*	*0.55*
*TNFRSF4*	01p36	**0.32**	**0.14**	*0.52*	*0.49*
*GNB1*	01p36.33	0.88	0.84	*0.43*	*0.45*
*TNFRSF14*	01p36.32	1.17	0.9	*0.48*	*0.48*
*TP73*	01p36.3	**0.14**	**0.11**	*0.61*	*0.57*
*PARK7*	01p36.33-p36.12	1	0.82	*0.59*	*0.57*
*MFN2*	01p36.22	1	1.02	1.24	1.3
*PTAFR*	01p35-p34.3	1.24	1.01	*0.57*	*0.55*
*FAF1*	01p33	1.2	1.01	*0.53*	*0.52*
*PPAP2B*	01pter-p22.1	**0.31**	**0.26**	*0.59*	*0.57*
*CYP2J2*	01p31.3-p31.2	1.28	1.04	*0.52*	*0.54*
*LPHN2*	01p31.1	1.16	0.98	*0.57*	*0.53*
*GTF2B*	01p22-p21	1.1	0.89	*0.59*	*0.59*
*DPYD*	01p22	**0.21**	**0.15**	*0.59*	*0.54*
*NRAS*	01p13.2	0.83	*0.64*	*0.63*	*0.57*
*LMNA*	01q22	1.18	0.91	1	1.05
*CRB1*	01q31.3	0.74	*0.4*	0.88	0.88
*TNNT2*	01q32.1	1.03	0.96	0.74	0.73
*SMARCA4*	19p13.2	1.06	1.08	1.3	1.39
*LDLR*	19p13.3	1.17	1.02	1.04	1.04
*CCNE1*	19q12	0.88	0.81	*0.57*	*0.58*
*PDCD5*	19q12-q13.1	**0.2**	**0.19**	*0.52*	*0.5*
*UPK1A*	19q13.13	1.14	0.79	*0.62*	*0.58*
*TGFB1*	19q13.1	0.95	0.83	*0.53*	*0.5*
*PPP1R15A*	19q13.2	0.67	*0.42*	*0.6*	*0.57*
*BAX*	19q13.3-q13.4	*0.5*	*0.36*	*0.58*	*0.58*
*CHMP2A*	19q13.43	0.97	0.85	*0.54*	*0.53*
Control	02p16	1.16	1.14	1.04	1.07
Control	03p25.3	1.35	1.15	0.88	0.83
Control	03q	1.07	1.02	1.06	1.05
Control	05q22	1.24	1.13	0.86	0.85
Control	08q	0.89	*0.66*	0.95	1
Control	08q13	1.23	0.98	1	1.01
Control	09q21	1.07	1.08	1.3	1.39
Control	11q23	1.34	1.09	1.05	1
Control	13q14.3	0.99	1	1.22	1.27
Control	14q	0.94	0.91	1	1
Control	14q22	0.99	1.01	1.23	1.28
Control	15q21.1	1	0.98	*0.56*	*0.56*
Control	17q11.2	0.88	*0.63*	0.89	0.92
Control	17q21	0.98	1	1.22	1.27

Genes are named by their official symbol. P correspond to the pediatric glioma of study and PC to an oligodendroglioma with known 1p/19q codeletion (positive control). Number are the values obtained by Coffalyser analysis, normalized with DNA from 4 healthy volunteers (references). Bold style represents total marker loss and italics Loss of Heterozygosity.

### Isocitrate dehydrogenase 1 and 2 (*IDH1/2*) mutation analysis

The genomic regions spanning the R132 codon of *IDH1* and R172 of *IDH2* genes were amplified and sequenced with an ABI PRISM 3100 Genetic analyzer and Sequencing Analysis 5.1.1 software (Applied Biosystem, Foster City, CA, USA) using the primers and conditions described previously [14,15].

## Results

**Figure 4:**
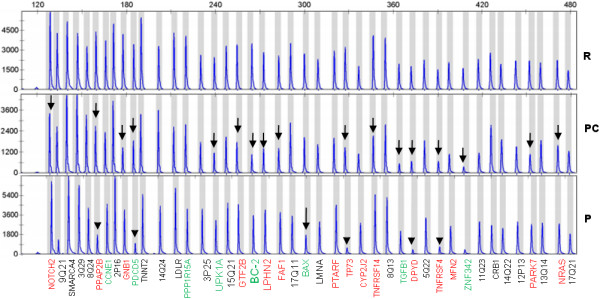
**MLPA electrophoresis peak-area patterns.** R: Reference non tumoral DNA. PC: Positive control of an oligodendroglioma with known 1p/19q codeletion (arrows). P: pediatric glioma with homozygous deletion of a number of markers (arrow-heads) and hemizygous deletion of *BAX* (arrow). Genes and genomic regions at 1p are coloured in red, at 19q are green, and the other probes are black.

The study was performed in duplicate and five control samples were used: one corresponding to a known oligodendroglioma with proven 1p/19q loss (positive control), and four non-tumoral DNAs (used as references for MLPA analysis). As shown in Figure 4 and Table 1, an almost complete absence of signal was identified in a few markers: *TNFRSF4* (located at 1p36.33), *TP73* (1p36.32), *PPAP2B* (1pter-p22), *DPYD* (1p21.3) and *PDCD5* (19q13.12), suggesting that total loss of these genomic regions occurred in the pediatric tumor reported herein. In addition, heterozygotic loss of *BAX* (19q13.3-q13.4) was present, and no mutations of *IDH1/2* were identified. Probe for *ZNF342* presented problems even in references and therefore, it was excluded from assay. Control probe 12p13 was also excluded because pediatric glioma and positive control showed a value of 0. Finally, the tumor DNA displayed allelic loss involving the 9q21 control marker, although this was not numerically confirmed (Table 1).

## Discussion and conclusions

Pediatric gliomagenesis seems to follow different molecular pathways from adults. Concurrent deletion of 1p and 19q is the hallmark of adult oligodendrogliomas (identified in up to 80% of tumors) [16-18] and results from an unbalanced t (1;19) (q10; p10) [19,20]. Assessment of allelic status at 1p and 19q has been established as a key tool for the diagnosis, treatment and prognosis of adult oligodendrogliomas, given that tumors carrying the co-deletion usually have a more favorable response to conventional chemotherapy agents [21,22]. Similarly, *MGMT* aberrant promoter methylation has been proposed as potentially associated with chemosensitivity and prolonged survival [23,24]. *IDH1* mutations present in most oligodendrogliomas, most astrocytic tumors and secondary glioblastomas have been associated with better overall survival and progression-free survival [15,25]. Aberrant promoter methylation has also been described [26] and up to three epigenetic oligodendroglioma groups with various clinical and biological features have been identified [27]. These neoplasms are characterized by inactivating mutations of *CIC*, concurrent with *IDH* sequence changes and 1p/19q deletions [28]. Oligodendrogliomas are less commonly present in children (1%-3% of pediatric CNS neoplasms) [3] and the available molecular data suggest that pediatric gliomas display molecular features that are slightly different from those present in adults [1,7,8], such as no *IDH1* or *TP53* mutations and positive for *MGMT* methylation. However, 1p/19q concurrent deletion appears rare, although it is present in oligodendrogliomas from older children (over 10 years of age) [29-31]. Correct identification of these molecular changes might be useful if a similar correlation with prognosis could be found in pediatric oligodendrogliomas. However no correlation between 1p/19q deletion and increased survival has been described in a series of high grade malignant pediatric gliomas, most of which were classified as astrocytomas [32]. It is possible that the molecular implications of losses involving 1p/19q in pediatric glial tumors (including oligodendroglial tumors) are different from those in adult cases, i.e., that different genes could be the targets of these alterations and thus no correlation with increased survival should be expected. Candidate genes at 1p/19q have been investigated in the search for potential targets of that co-deletion, including *NOTCH2*, *CDKN2C*, *RAD54*, *CITED4*, *CAMTA1*, *TP73*, *EMP3* and *PEG3*, but no consistent mutation of these genes has been demonstrated [8,33-35]. The infrequent presentation of oligodendrogliomas in pediatric patients and the unconventional microscopic pattern observed in the case reported herein could justify the generic diagnosis (anaplastic glioma). However, a number of other microscopic features (capillary network, micro-calcifications, CD57 positivity) are consistent with the presence of an oligodendroglioma component. The molecular findings in this tumor, which demonstrate 1p/19q co-deletion (including homozygosus deletion of a few markers) could also support an oligodendroglioma diagnosis, although these molecular alterations are rare in pediatric oligodendrogliomas [30]. The absence of *IDH1* mutations also agrees with the data in pediatric gliomas, including oligodendrogliomas [36,37]. Recent reports have identified no *IDH1* mutations with variable allelic constitutions for 1p/19q (including co-deletion to no loss) in disseminated oligodendroglial-like leptomeningeal tumors in pediatric patients [38,39]. However, no apparent leptomeningeal involvement was evident in the case reported herein. Another report described an aggressive clinical phenotype (8-month overall survival) characterizing a pediatric glioblastoma with an oligodendroglioma component, displaying neither *IDH1/2* mutation nor 1p/19q co-deletion [40], which was clearly different from the case reported here. In conclusion, a definitive histopathological diagnosis could not be clearly established, and the doubt remains about whether this tumour could be classified as displaying (or not) a true oligodendroglial component. However, the identification of homozygous deletion involving a number of 1p/19q markers provides insights on genomic regions in these chromosome arms, where potential critical target genes, which could participate in the development of pediatric (oligodendro) gliomas, might be located. As shown in Table 2, several genes involved in interesting biological functions are located in the neighborhood of the homozygous deleted markers in the case reported here. Functional and/or mutational analyses of these neighbour genes would provide insights on potential new regulation pathways, perhaps non-randomly involved in the development of pediatric gliomas.

**Table 2:** Biological process of altered genes identified by MLPA in pediatric glioma and of neighbour genes located in the critical deleted regions

**Gene**	**Biological process**	**Chromosomal region**	**Neighbor genes**	**Biological process**
*TNFRSF4*	Inflammatory and immune response	01p36	*TNFRSF18*	Apoptosis
			*TTLL10*	Protein polyglycylation
			*SDF4*	UV protection
*TP73*	DNA damage response	01p36.3	*ARHGEF16*	Apoptosis
			*LRRC47*	Translation
			*SMIM1*	
			*WRAP73*	
			*TPRG1L*	Synapse
			*MEGF6*	Calcium ion binding
*PPAP2B*	Blood vessel development	01pter-p22.1	*PRKAA2*	Protein phosphorylation
			*C1orf168*	
*DPYD*	Purine base catabolic process	01p22	*PTBP2*	mRNA processing
*PDCD5*	Apoptosis	19q12-q13.1	*ANKRD27*	Early endosome to late endosome transport
			*DPY19L3*	
			*CEP89*	
			*SLC7A9*	Protein complex assembly
			*TDRD12*	
			*NUDT19*	
			*RGS9BP*	Negative regulation of signal transduction
*BAX*	Apoptosis	19q13.3-q13.4	*FTOL*	
			*NTN5*	
			*PP1R15A*	Apoptosis
			*NUCB1*	
			*CD37*	Regulation of humoral immune response
			*PPFIA3*	Neurotransmitter secretion
			*CGB*	
			*TYS1*	Apoptosis
			*DHDH*	

Only the most nearby protein-coding genes were selected.

## Consent

Written informed consent was obtained from the parents for publication of this case report. A copy of the written consent is available for review by the Editor-in-Chief of this journal. Also, the molecular studies were performed on anonymized samples provided to the Molecular Neuro-Oncogenetics laboratory (IdiPAZ).

## Competing interests

The authors declare that they have no competing interests.

## Authors’ contributions

MT-M and CP-G performed MLPA analysis. BM performed *IDH* mutation studies. FC performed surgery and MG performed histo-pathological studies, and both of them collected the clinical data relative to this case. RRB, GRP and JSC provided tools for analysis and analyzed the findings. MT-M, BM and JAR analyzed the findings, drafted the paper and revised the manuscript for important intellectual content. All authors contributed to the finalizing of the manuscript. All authors read and approved the final manuscript.
